# Approaches to neonatal intubation training: A scoping review

**DOI:** 10.1016/j.resplu.2024.100776

**Published:** 2024-09-23

**Authors:** Jasmine Antoine, Brian Dunn, Mia McLanders, Luke Jardine, Helen Liley

**Affiliations:** a Mater Mothers’ Hospital, Mater Research and The University of Queensland, Australia; b Joan Kirner Women's and Children's, Sunshine Hospital & The University of Queensland, Australia; c Clinical Skills Development Service, Metro North and The University of Queensland, Australia; d Mater Mothers’ Hospital, and The University of Queensland, Australia

**Keywords:** Newborn, Infant, Teaching, Resident medical officers, Resuscitation, Laryngoscope, Tracheal intubation

## Abstract

**Introduction:**

Neonatal intubation is a lifesaving skill that a variety of clinicians need to establish as it can be required anywhere babies are born or hospitalised and cannot depend on the immediate availability of an experienced senior clinician. However, neonatal intubation is complex and risky, requiring technical and non-technical skill competence. Studies report that rates of successful neonatal intubation by junior clinicians are low, providing a mandate to examine the best methods to improve skill acquisition, retention, and transfer.

**Method:**

We utilised PRISMA-ScR methodology to capture the range of training approaches in the simulation and clinical settings, and to assess the range of technical and non-technical skill outcome measures that were used in the included studies. Databases were searched from inception to August 2024 to identify studies reporting outcomes for medical practitioners-in-training, nurses, and nurse practitioners. Identified studies meeting inclusion criteria underwent data charting with study characteristics tabulated.

**Results:**

Twenty-six studies (involving 1449 participants) were included. Training methodology was diverse and included self-directed learning, didactic education, demonstration, simulation-based training (SBT), instructor feedback, debriefing and supervised clinical practice. Most of the studies (96 %) used multiple training methods with education and SBT most frequently used. Thirteen studies reported outcomes in clinical settings, including seven that demonstrated changes in technical skills following education and SBT. Two studies that assessed transfer of skills failed to show successful transfer from simulation to a clinical setting. Two articles reported the transfer of skills between direct and video laryngoscope devices. Only one study evaluated skill retention (at 6–9 months) but did not demonstrate proficiency after initial training or at follow up. No studies described the effects of training on non-technical skills.

**Conclusion:**

No included studies or combination of studies seems likely to provide a high-certainty evidence-basis for optimal training methodology. Results suggested using a training bundle including education, SBT and supervision. Knowledge gaps remain, including the most effective methodology for non-technical skill training. In addition, the evidence of technical skill retention beyond the immediate training episode, and transfer to a variety of clinical environments is very limited. Given the importance of successful neonatal intubation, more research in these areas is justified.

## Highlights

• Education and simulation-based training are the most frequently used components of training bundles for neonatal intubation, but may not suffice without a structured phase of supervised clinical practice.
• All included studies approached neonatal intubation as a technical skill, and none addressed non-technical skills that are essential for optimal patient outcomes.
• Retention and transfer of technical skills in neonatal intubation is poorly researched.

## Introduction

### The importance of neonatal intubation training

Neonatal intubation is a lifesaving task that can be needed in a variety of clinical settings.^1^ It is a complex task with multiple steps requiring competence in technical and non-technical skills.^2^, ^3^ ‘Technical skills' describes psychomotor ability. ‘Non-technical skills' describes leadership, teamwork, and communication. Adverse events occurring in up to 50 % of patients include oxygen desaturation, bradycardia, mucosal trauma, and failed intubation.^4^, ^5^, ^6^ Safer neonatal intubation requires improving both technical and non-technical skills.^7^, ^8^.

Mastery of clinical skills includes three distinct phases; skill acquisition, measured as having the skills to perform the task, retention (recall), measured at a time weeks or months later, and transfer, the ability to perform the acquired skills in varied environments, team members, or key equipment. Comprehensive training in neonatal intubation results in skill acquisition, retention, and transfer.

### Why focus on the training of novice clinicians?

Less experienced clinicians have worse patient-centred outcomes,^9^, ^10^, ^11^, ^12^ with junior clinicians succeeding on fewer than 50 % of first attempts.^4^, ^10^ However, most locations cannot rely on the availability of a senior clinician for all neonatal intubations. Clinical opportunities to acquire proficiency are diminishing^13^, ^14^ for reasons including increased use of non-invasive respiratory support, and abandonment of routine intubation at delivery for airway suctioning of apnoeic babies exposed to meconium.^13^, ^15^, ^16^, ^17^, ^18^.

### Is any specified training approach better than any other approach?

Methods of neonatal intubation training for novice clinicians include self-directed learning, didactic education, simulation-based training (SBT), instructor feedback, supervised clinical practice and debriefing.^19^ While each of these has a role for training in complex clinical tasks, there is little evidence verifying which is best for skill acquisition, retention and transfer.

For this review we use the term education to describe didactic education provided to an individual or small group, with or without slide presentations or video-recordings or via an application. SBT describes skill stations or immersive simulation events with manikins or part task trainers. SBT may include instructor feedback during the simulation. Debriefing occurs either after a simulation or clinical session.

### Is it better to use SBT, or training in a clinical setting?

SBT is now widely used in the training of neonatal intubation.^20^ The benefits include opportunities to learn and practice without risk to patients and opportunities for debriefing after the simulation.^21^, ^22^, ^23^ However, constraints include potential lack of realism related to fidelity of manikins, scenarios, level of clinician stress and environmental complexity.^24^.

### What are the important outcomes measures of training in neonatal intubation?

Pragmatic measures include the proportion of successful intubations on the first attempt, overall success, number of attempts and time taken to intubate. The time to intubate is often measured from laryngoscope blade insertion to placement of the endotracheal tube. A more patient-centred measure is time from cessation of non-invasive ventilation to commencement of effective ventilation via the (correctly placed) endotracheal tube.^25^ Other patient-centred measures include adverse events, desaturation, bradycardia, or mucosal and airway trauma. Non-technical skills during intubation are also required to optimise patient outcomes.^7^, ^26^, ^27^ Although deficiencies in non-technical skills contribute to errors and adverse events,^8^, ^28^ few studies in the clinical training literature report these outcomes.

### Does video laryngoscopy compared to direct laryngoscopy enhance training?

Video laryngoscopy use in neonatal intubation training is increasing.^29^ Video laryngoscopy allows supervisors to see what the learner is seeing to offer real-time guidance, and to store recordings for debriefing.^30^, ^31^ However, video laryngoscopy differs from direct laryngoscopy, with potential impact on skill transfer between different video laryngoscope devices and to or from direct laryngoscopes.^30^.

### Do enhancements such as virtual reality augmented training or cognitive aids improve outcomes?

Virtual reality augmented training includes headsets to create immersive environments or overlay of digital information in SBT.^32^ Virtual reality training has been demonstrated to improve clinician’s technical and non-technical skills for neonatal procedures.^32^.

Cognitive aids include algorithms, checklists, and decision-making tools, can improve clinician and team performance.^33^ Poorly designed cognitive aids may impede performance if they do not include pertinent information, or if pathways are difficult to follow.^33^.

## Aim

We aimed to synthesise the literature on neonatal intubation training methods for novice clinicians, using comprehensive scoping review methods. Specific aims were to assess whether there was evidence for training settings, methodologies, devices, or paradigms to improve skill acquisition, retention and transfer in the simulation or clinical setting. Outcomes measures of interest were technical and non-technical skills.

## Method

This scoping review was conducted using the Arksey and O’Malley^34^ framework for identification of the research question, study search, study selection and data charting and is reported according to the PRISMA-ScR format.^35^ The PRISMA-ScR checklist is detailed in Appendix 1. The protocol was registered Open Science Framework https://osf.io/75btk/)

### ***Search*** strategy***, inclusion criteria and data sources***

The search strategy and PICOST are described in Appendix 2 and 3. Appendix 1 and 3 Eligible studies included randomised, quasi-randomised controlled, cohort, prospective and retrospective trials that described intubation training for junior medical officers (excluding consultants or attendings), nurses, nurse practitioners (NP) and NP candidates. Mixed methods, qualitative research, and published quality improvement projects were also included. Studies that did not present original data on the effectiveness of the training program for improving technical or non-technical skills in neonatal intubation were excluded. The databases searched were PubMed, EMBASE, the Cochrane Central Register of Controlled Trials, Google Scholar and CINAHL. The search was confined to studies published in English to 21st August 2024.

### ***Study*** selection

After removing duplicates, two independent reviewers (JA, BD) screened titles and abstracts for eligibility using Covidence software (Melbourne, Australia) and selected full-text articles for review. Reference lists of included studies were also hand-searched for further eligible studies. Conflicts were resolved by consensus or a third reviewer (HL).

### ***Data extraction*** and ***charting***

Data extraction and charting were performed independently by two authors (JA, BD). A standardised data charting form was utilised to systematically record study characteristics, methodology, outcomes and key results (Appendix 4- Detailed tables of included studies). This process ensured consistency. Confirmation of the data charting was performed by the other author (JA, BD).

## Results

### ***Search*** and ***study selection***

**Fig. 1:**
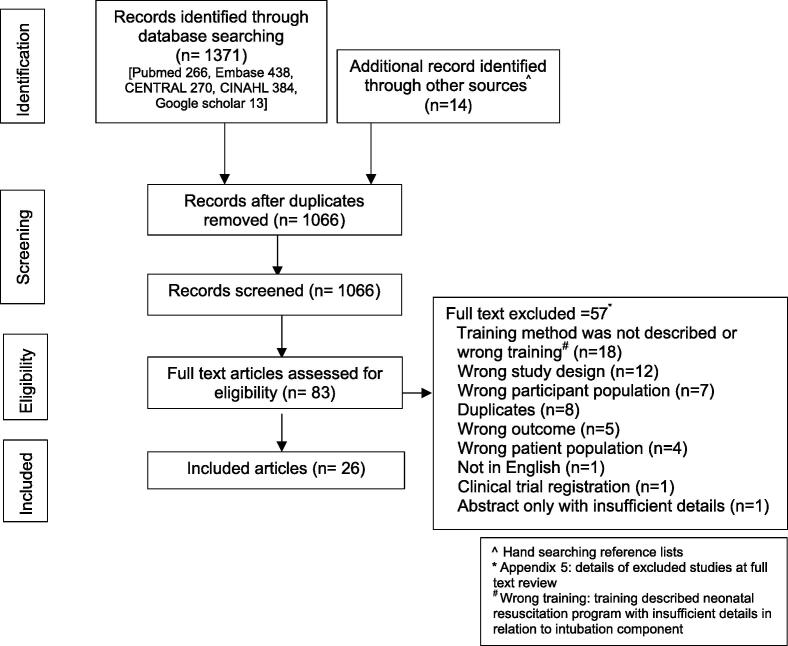
PRISMA.

The search identified 1066 unique records from which 83 articles were screened in full text, and 26 articles with 1449 participants were included (Fig. 1). Appendix 5 summarises reasons for excluding articles at full text review.

### ***Study*** characteristics

**Fig. 2:**
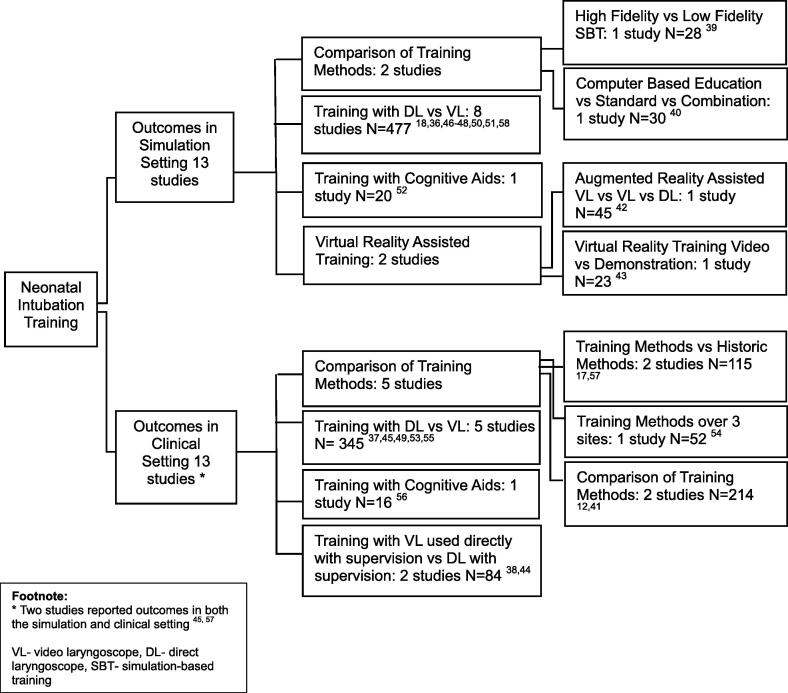
Network Map of Studies.

Included studies had varied study design, participant demographics, training intervention, device intervention and outcome measures. Due to the heterogeneity, studies were grouped by intervention and the setting in which outcomes were measured; simulation, clinical or both (Appendix 4). A network map of studies is shown in Fig. 2.

Of included studies, 9 were randomised controlled trials (RCTs),^36^, ^37^, ^38^, ^39^, ^40^, ^41^, ^42^, ^43^, ^44^, ^45^ 7 were randomised cross-over studies,^18^, ^46^, ^47^, ^48^, ^49^, ^50^ and one used a quasi-experimental design.^12^ One study used a non-randomised cross-over design.^51^ The remaining studies were observational.^17^, ^52^, ^53^, ^54^, ^55^, ^56^, ^57^, ^58^.

The studies came from 9 countries (Appendix 6), with the largest numbers from the USA (n = 11) and Canada (n = 6). All studies involved medical officers-in-training, nurses, and nurse practitioners. Studies that included respiratory therapists, paramedics, advanced practice clinicians, attendings and consultants were included if novices were also involved (Appendix 7).

### ***Types of*** training

**Fig. 3:**
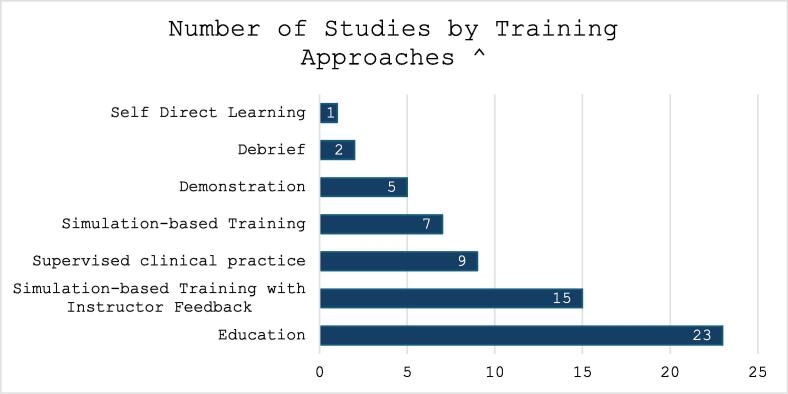
**Number of Studies by Training Approach***Footnote:^Training approaches were not mutually exclusive, most studies had more than one approach.*

Training approaches studies were diverse (Fig. 3) and included self-directed learning, didactic education in person and video, demonstration of skills, SBT with and without feedback, supervised clinical practice, and debriefing. All but one study used more than one approach.^52^ Notably, education was used in 23 studies, and SBT in 22 studies. Education-based training used either slide or video presentations with or without a trainer. Education content included airway anatomy, indications for intubation, equipment, procedure, troubleshooting, and complications. Some also incorporated crisis resource management and escalation pathways for difficult airway management. Self-directed learning modules had similar content.

Of the 22 studies that used SBT,^12^, ^17^, ^18^, ^50^, ^36^, ^37^, ^38^, ^39^, ^40^, ^41^, ^42^, ^44^, ^45^, ^46^, ^47^, ^48^, ^53^, ^54^, ^55^, ^56^, ^57^, ^58^ 15 included feedback during the SBT. SBT included skill stations and immersive scenarios. The training used part-task trainers, low-fidelity or high-fidelity manikins from equivalent to 25-weeks’ gestation preterm to 6-month-old term infants.

Nine studies incorporated supervised clinical practice: 5 used demonstration of skills and 3 included debriefing. Debriefing occurred with or without video laryngoscope recordings.

### ***Duration of*** training

Fifteen studies detailed duration of training curriculum, which varied from a single 5-minute episode^41^ to one month of repeated training sessions in the operating theatre.^53^ Of the studies that reported training session duration, six demonstrated improvement in outcomes after training of 60 min or less.^18^, ^41^, ^47^, ^48^, ^50^, ^52^ However, four other studies of short-duration training did not show benefits,^12^, ^36^, ^43^, ^46^ and no studies specifically compared short- and longer-duration training.

### ***Training*** outcomes

**Fig. 4:**
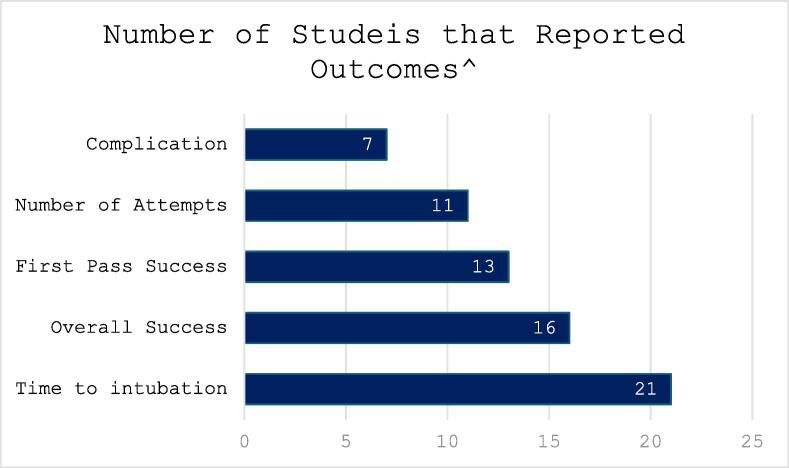
Number of Studies that Reported Outcomes.

Technical skills outcomes were reported in all studies. The most frequent outcome measures reported in studies were time to intubation (n = 21), overall success (n = 16), first pass success (n = 13), number of attempts (n = 11) and complications (n = 7) (Fig. 4). Training outcomes were defined as per author’s definitions. Definitions of time to intubation varied. Reported complications included episodes of desaturation, bradycardia, mucosal trauma, and oesophageal intubations.

Training outcomes were measured in simulation (13 studies) or clinical (11 studies) settings, while two studies reported training outcomes in both.^45^, ^57^ Training outcomes in clinical settings were measured in neonatal units (n = 8), during retrieval or patient transport (n = 3), in the birth suite or obstetric operating theatre (n = 3), and the operating theatre before neonatal surgery (n = 1). No studies specifically compared patient outcomes from training in simulation settings to those in of training in clinical settings.

### Studies comparing training methods

Seven studies (439 participants) compared training methods. Of the two studies reporting outcomes in simulation (Table 1),^39^, ^40^ one study compared high versus low fidelity simulators, and found no difference pre- and post-training, or between simulators.^39^ There were also no differences between groups 6–9 months after training. A second study compared training with or without computer-based education with SBT and demonstration, and showed faster time to successful intubation after any training intervention using either direct or video laryngoscopy, but there was no difference between training interventions.^40^.

**Table 1:** Studies Comparing Training Methods and Measuring Simulation Outcomes.

**Author** **Year** **Country**	**Study Design**	**Participants**	**Primary Intervention**	**Control**	**Training** **Setting**	**Outcome Setting**	**Outcome**		
							**First Pass Success**	**Overall Success**	**Time to Intubation**
Al-Wassia 2022 Saudi Arabia	RCT	28 junior doctors	Ed + SBT with high fidelity manikin + instructor feedback	Ed + SBT with low fidelity manikin + instructor feedback	Sim	Sim	N/A	Before to after training Between training groups	Before to after training Between training groups
Koele-Schmidt 2016 USA	RCT	30 junior doctors	1. Ed computer based + SBT Ed computer based + SBT+standard teaching	3. SBT+Standard teaching: didactic ed + demo	Sim	Sim	N/A	N/A	Before to after training Between training groups

**Footnote:**

Abbreviations: RCT; randomised controlled trial, Ed; education, SBT; simulation-based training, Demo; demonstration, Sim; simulation

Positive effect.  No effect. Negative effect. N/A Not assessed.

Of the five studies reporting clinical outcomes (Table 2),^12^, ^17^, ^41^, ^54^, ^57^ two studies compared outcomes for learners exposed to a training bundle with a historic cohort.^17^, ^57^ Two studies compared different training methods using a randomised^41^ or quasi-randomised design,^12^ and one had no control.^54^ All the training bundles included education and SBT. All included instructor feedback and three included supervised clinical practice. Two of these studies found benefit (Table 2).

**Table 2:** Studies Comparing Training Methods and Measuring Clinical Outcomes.

**Author** **Year** **Country**	**Study Type**	**Participants**	**Primary Intervention**	**Control**	**Training** **Setting**	**Outcome Setting**	**Outcome**		
							**First Pass Success**	**Overall Success**	**Time to Intubation**
Akierman 2002 Canada	Observational retrospective	52 doctors*, RT& RN	Training: Ed + SBT+instructor feedback + supervised clinical practice	Nil	Sim + clin	Clin	N/A		N/A
Finan 2012 Canada	Observational	13 junior doctors	Training: Ed + demo + SBT+instructor feedback	Historic cohort-no training	Sim	Sim + Clin	N/A		
Gizicki 2023 Canada	RCT	112 junior doctors	Just in time training: Ed with DL/VL+SBT+instructor feedback	Video education- Ed with DL/VL	Sim	Clin			
O’Shea 2021 Australia	Observational	102 doctors*, NNP	Ed + SBT with DL/VL+instructor feedback + procedure pause + supervised clinical practice + VL introduction	Historic cohort	Sim + clin	Clin			N/A
Rumpel 2022 USA	qRCT	102 junior doctors	Intervention training program 30-minute Ed + SBT using premature Anne manikin with VL used directly + one- one instructor feedback + repeat SBT intubation time < 15 s + standard training	Standard training: 15 min SBT on term part task manikin with DL+instructor feedback + supervised clinical practice	Sim + Clin	Clin			N/A

**Footnote:**

Abbreviations: RT; respiratory therapist, RN; registered nurse, NNP; neonatal nurse practitioners, DL; direct laryngoscopy, VL; video laryngoscopy, RCT; randomised controlled trial, qRCT; quasi randomised controlled trial, SDL; self-directed learning, Ed; education, Demo; demonstration, SBT; simulation-based training, Sim; simulation, Clin; clinical.

Positive effect.  No effect.  Negative effect N/A Not assessed.

* Includes junior and senior medical officers,

Skill transfer from the simulation to the clinical environment was investigated in one study.^57^ This study investigated the effect of didactic education and SBT on participants’ intubation performance and found that an improved global rating scale in the simulation setting was not sustained in the clinical environment.

### ***Training with*** video laryngoscopy ***compared to direct laryngoscopy***

Thirteen studies compared video laryngoscopy to direct laryngoscopy. Eight studies (477 participants) reported outcomes in the simulation setting (Table 3),^18^, ^36^, ^50^, ^51^, ^58^, ^46^, ^47^, ^48^ and 5 studies (345 participants) in clinical settings (Table 4).^37^, ^45^, ^49^, ^53^, ^55^ Outcomes in simulation settings were inconsistent. In clinical settings, video laryngoscopy resulted in improved success and lower complication rates for novice clinicians.

**Table 3:** Training with Video Laryngoscopy Compared to Direct Laryngoscopy Measured in Simulation.

**Author** **Year** **Country**	**Study Design**	**Participants**	**Primary Intervention**	**Control**	**Training Setting**	**Training Method**	**Outcome Setting**	**Outcome**		
								**First Pass Success**	**Overall Success**	**Time to Intubation**
Grgurich 2016 USA	RCT (cross over)	19 nurses	VL	VL used directly with video output covered	Sim	Ed + SBT	Sim	N/A	N/A	
Johnston 2014 USA	RCT (cross over)	64 doctors*	VL	DL	Sim	Ed + SBT	Sim	N/A	N/A	Residents DL faster VL Fellows
Komasawa 2015 Japan	RCT (cross over)	23 junior doctors	VL+chest compression	DL+chest compression	Sim	SBT+instructor feedback	Sim	N/A	Without chest compression With chest compression	
Musharaf 2020 Canada	Non-randomised (cross over)	26 doctors*, RT, transport nurses, NNP	VL	DL	Sim	Ed + demo	Sim		N/A	Junior doctors, RT, nurses Physicians, NNP
Nair 2017 USA	RCT	64 junior doctors & 59 RT	VL with training in DL/VL	DL with training in DL only	Sim	Ed + SBT+instructor feedback	Sim		N/A	
Parmekar 2017 USA	RCT (cross over)	100 junior doctors	VL with VL training	DL with DL training	Sim	Ed + SBT+instructor feedback	Sim		VL trained group with VL vs DL DL trained group with VL vs DL	VL trained vs DL trained on FPS VL trained group faster with DL DL trained group with VL
Shaylor 2023 Israel	RCT (cross over)	23 junior doctors	CMAC VL+McGrath VL+difficult airway manikin	DL+difficult airway manikin	Sim	Ed + SBT+instructor feedback	Sim	N/A	VL to DL Between VL groups	DL faster than VL Between VL groups
Zhou 2020 China	Observational	62 nurses & midwives, 37 doctors*	VL visible	DL	Sim	Demo + SBT+instructor feedback	Sim	N/A	Experience Less experience Novice	Experience Less experience Novice

**Footnote:**

Abbreviations: RCT; randomised controlled trial, VL; video laryngoscopy, Sim; simulation, Ed; education, SBT; simulation-based training, Demo; demonstration, DL; direct laryngoscopy, RT; respiratory therapist, NNP; neonatal nurse practitioners.

Positive effect.  No effect.  Negative effect N/A Not assessed.

* Doctors- combination of junior and senior medical officers.

**Table 4:** Training with Video Laryngoscopy Compared to Direct Laryngoscopy and Measuring Clinical Outcomes.

**Author** **Year** **Country**	**Study Design**	**Participants**	**Primary Intervention**	**Control**	**Training Setting**	**Training Method**	**Outcome Setting**	**Outcome**			
								**First Pass Success**	**Overall Success**		**Time to Intubation**
Abid 2021 USA	Observational retrospective	123 RN, 12 paramedics	VL	DL	Sim + Clin	Ed + SBT+instructor feedback + supervised clinical practice	Clin		N/A	N/A	
Coutu 2022 Canada	Observational	67 RN, 30 RT, 6 doctors*	VL	DL- (historical cohort group)	Sim + Clin debrief	Ed + SBT+debrief recorded clinical intubations	Clin			N/A	
Moussa 2016 Canada	RCT (cross over) Phase 1: randomised to VL vs DL Phase 2: Both VL and DL group used DL	34 junior doctors	VL	DL	Sim + Clin	Ed + SBT+instructor feedback + supervised clinical practice	Clin	N/A	Phase 1 VL vs DL Phase 2 VL to DL Phase 2 DL to DL		
Saran 2019 India	RCT (cross over)	24 junior doctors	VL+feedback	VL used directly + feedback	Clin	Ed + supervised clinical practice during testing	Clin		N/A		
Yankowski 2022 USA	RCT	47 junior doctors, 2 advance practice clinicians	VL visible + DL training	DL training	Sim	Ed + SBT+instructor feedback	Sim + Clin	Sim setting		Sim setting	

Footnotes:

Abbreviations: FPS; first pass success, VL; video laryngoscopy, DL; direct laryngoscopy, Sim; simulation, Clin; clinical, Ed; education, SBT; simulation-based training, RCT; randomised controlled trial, RN; registered nurse, RT; respiratory therapist.

Positive effect.No effect.  Negative effect, N/A Not assessed.

* Some doctors in this study were not novices.

In the simulation setting the use of video laryngoscopy did not offer a consistent advantage over direct laryngoscopy for novice practitioners. All studies evaluated time to intubation; two of eight found no difference between groups,^36^, ^46^ three had varied results depending on the participant subgroups.^18^, ^51^, ^58^ One study showed faster intubation time with video laryngoscopy^47^ and one study slower time to intubation with video laryngoscopy than direct.^50^ Interestingly in a randomised cross-over study, Parmekar, Arnold^48^ initially showed no difference in time to intubation between devices, but following cross-over, those participants who started with video laryngoscopy were faster to intubate with direct laryngoscopy.^48^ They also found that skills learned with video laryngoscopy could be transferred to direct laryngoscopy without further training. Of the four studies reporting overall success of intubation, results were mixed.

The multi-phase study by Moussa, Luangxay^37^ participants were trained with direct laryngoscopy in simulation, followed by randomisation to either direct or video laryngoscopy in the clinical setting. In phase two, all participants then intubated in the clinical setting with direct laryngoscopy. There was higher overall success in the group randomised to use video laryngoscopy than direct laryngoscopy, but no difference in overall success in phase two when participants were tested in the clinical setting with direct laryngoscopy.^37^.

Skills transfer between simulation and clinical settings was investigated in one study.^45^ Yankowski, Kovatis^45^ investigated the effect of video laryngoscopes, education, SBT with instructor feedback, and found no difference between junior doctor groups in the simulation or clinical setting.^45^

### ***Is intubation improved when*** supervisors ***can see the airway on a*** video ***screen?***

Two studies (84 participants) randomised learners to direct laryngoscopy with a supervisor who either was or was not able to see the airway via a video screen (Table 5).^38^, ^44^ First pass success was improved in both studies when the supervisor was able to view the video display of the airway during intubation,^38^, ^44^ and Volz, Stevens^38^ reported improved overall success. There was no difference in the time to intubation or rates of complication.^38^, ^44^.

**Table 5:** Studies Comparing Training with Video Laryngoscopes used Directly with Supervision to Direct Laryngoscopy with Supervision (Clinical Outcomes).

**Author** **Year** **Country**	**Study Design**	**Participants**	**Primary intervention**	**Control**	**Training** **Setting**	**Training method**	**Outcome Setting**	**Outcome**		
								**First pass success**	**Overall success**	**Time to intubation**
O'Shea 2015 Australia	RCT	36 junior doctors	VL with learner using direct view, supervisor able to see airway via VL display	VL used directly with supervisor unable to see VL output	Sim + Clin	Ed + SBT+supervised clinical practice + debrief clinical intubations	Clin		N/A	
Volz 2018 USA	RCT	48 junior doctors	VL with learner using direct view, supervisor able to see airway via VL display	DL	Sim + Clin	SDL+Ed + SBT+supervised clinical practice	Clin			

**Footnote:**

Abbreviations: RCT; randomised controlled trial, VL; video laryngoscope, DL; direct laryngoscope, Sim; simulation, Clin; clinical, Ed; education, SDL; self-directed learning.

Positive effect.  No effect  Negative effect. N/A Not assessed.

### ***Virtual*** reality ***assisted training***

Virtual reality assisted training with outcomes in the simulation setting was investigated in two studies (68 participants) with varying outcomes (Table 6).^42^, ^43^ Dias, Greenberg^42^ undertook a RCT of three different intubation devices, together with instructor feedback and debriefing. Participants intubated either with virtual reality glasses projecting the airway, video laryngoscopy or direct laryngoscopy. Overall success and time to intubation improved for those who used virtual reality glasses or video laryngoscopy.^42^ O’Sullivan, Bosley^43^ compared training video using virtual reality to in-person skills demonstration and found no difference in overall success or time to intubation between groups.

**Table 6:** Virtual Reality Assisted Training.

**Author** **Year** **Country**	**Study Design**	**Participants**	**Primary Intervention**	**Control**	**Training** **Setting**	**Training Method**	**Outcome Setting**	**Outcome**		
								**First Pass Success**	**Overall Success**	**Time to Intubation**
Dias 2021 USA	RCT	45 nurses	1. Augmented reality assisted VL VL	DL	Sim	Ed + instructor feedback during testing + debrief	Sim	N/A		
O’Sullivan 2022 USA	RCT	23 clinicians*	Virtual reality training video	Demonstration	Sim	Ed or demonstration	Sim	N/A		

**Footnote:**

Abbreviations: RCT; randomised controlled trial, VL; video laryngoscopy, DL; direct laryngoscopy, Sim; simulation, Ed; education

Positive effect.  No effect.  Negative effect N/A Not assessed.

* “multidisciplinarhy trainees and clinicians”.

### Cognitive aid assisted training

Cognitive aids supplementing training improved neonatal intubation in both simulation and clinical settings. One article investigated a cognitive aid as part of SBT (20 participants) (Table 7),^52^ and another described the use of a cognitive aid in a clinical setting (16 participants) (Table 8).^56^ There were no studies that compared the effectiveness of different cognitive aids.

**Table 7:** Cognitive Aids with Outcomes Measured in Simulation.

**Author** **Year** **Country**	**Study Design**	**Participant**	**Primary Intervention**	**Control**	**Training Setting**	**Training Method**	**Outcome Setting**	**Outcome**		
								**First Pass Success**	**Overall Success**	**Time to intubation**
Hawkes 2013 Ireland	Observational	20 junior doctors	NeoTube education application & quick reference aid	Nil	Sim	Ed via application	Sim	N/A	N/A	

**Footnote:**

Abbreviations: Sim-;simulation, Ed; education

Positive effect.  No effect.  Negative effect N/A Not assessed.

**Table 8:** Cognitive Aid with Clinical Outcomes.

**Author** **Year** **Country**	**Study Design**	**Participants**	**Primary Intervention**	**Control**	**Training Setting**	**Training Method**	**Outcome Setting**	**Outcome**		
								**First Pass Success**	**Overall Success**	**Time to Intubation**
Dalrymple 2022 Australia	Observational	16 junior doctors	SBT+checklist	Historic cohort	Sim	SBT+checklist + supervised clinical practice	Clin		N/A	N/C

**Footnote:**

Abbreviations: SBT; simulation-based training, Sim; simulation, Clin; clinical.

Positive effect.  No effect.  Negative effect N/A Not assessed N/C no comparator.

## Discussion

Neonatal intubation training studies varied in their design, comparators and outcome measurements. Due to this heterogeneity, we were unable discern an optimal training method for neonatal intubation skills training for novice clinicians, or to identify a group of studies suitable for further systematic review and *meta*-analysis*.* A previous, more limited systematic review of instructional methods, assessment tools and training models in neonatal and paediatric intubation training also found no one method of intubation training was most effective.^59^ Importantly, we found that there was insufficient evidence for technical and non-technical skill outcomes that encompassed the full learning process: acquisition, to retention, to transfer.

### ***Types of*** training

This scoping review examined evidence for training approaches in neonatal intubation for novice clinicians including combinations of self-directed learning, education, SBT, instructor feedback, debriefing and supervised clinical practice. Since learning styles vary, ideal educational approaches should be flexible, and learner focused.^60^, ^61^.

We found some evidence that short duration training (<60 min) can result in skill acquisition. This is consistent with evidence that training as short as 2 min can enhance paediatric CPR skills.^62^ However, no studies compared different durations of training sessions and therefore, the optimal duration of training to improve skill acquisition, transfer, retention, and overall patient outcomes remains unknown.

### ***Training ou***t***comes***

In this review the effect of training was most frequently reported as technical skills acquisition, but *meta*-analysis was precluded by the varied study designs, outcome measures and definitions. For example, definitions of time to intubation included time from blade in mouth till time out of mouth, time from blade in mouth until confirmation of ETT placement or it was not defined at all. Coauthors of the current study previously showed using video recordings of intubation in the delivery room, that the time from cessation of ventilation via face mask to recommencement via a correctly placed ETT was often twice as long as the interval from blade in mouth to blade out, and they highlighted that this longer interval may be a more patient-centred measure of time to intubation, since neonates with longer intubation attempts are more likely to become bradycardic and desaturate.^25^ Another study confirmed that the time taken for clinical assessment to confirm endotracheal tube placement was often longer than duration of the intubation attempt itself.^11^ Several included studies reported times to successful intubation. However, there was insufficient information to address potential improvements in patient stability that might result from longer intervals of face mask ventilation between attempts.

Non-technical skills are essential for successful intubation and better patient outcomes.^26^, ^63^, ^64^, ^65^, ^66^ However, in this review no studies reported on the effect of training on non-technical skill outcomes such as teamwork, leadership and communication. In anaesthetics, errors in non-technical skills are associated with up to 80 % of adverse events.^28^ A systematic review found that teaching teamwork as part of neonatal resuscitation training may increase teamwork behaviour and reduce duration of resuscitation.^20^ Similarly, in the assessment of the neonatal resuscitation training program NeoSim™®, focused on technical and non-technical skill acquisition, communication skill development was rated by participants as the most influential for future neonatal resuscitation management.^67^ These findings support the need to assess non-technical skills outcomes in future studies.

### Comparison of training methods

SBT was one of the most-widely used training methods. However, it was never used as a sole intervention, or compared head-to-head with other methods, such as supervised clinical practice. This may be because the advantages of SBT for introducing novices to high-risk procedures are already well-recognised.^20^ We suggest SBT should be part of a comprehensive training package, and we found eight studies suggesting that SBT improves outcomes in a clinical setting,^17^, ^37^, ^38^, ^41^, ^44^, ^45^, ^55^, ^56^ with the caution that most of the remaining studies using SBT did not measure clinical outcomes. Transfer from SBT to clinical settings cannot be assumed. Only two studies reported skill outcomes in simulation then clinical settings^45^, ^57^ and both failed to demonstrate that improved performance in the simulation setting was transferred to the clinical setting. Reasons could include difficulties ensuring sufficient authenticity of simulation, due to issues including the anatomy and fidelity of manikins, and differences in clinician stress and complexity of scenarios.^24^.

The other widely used training method was education (23 studies), presented in various forms including didactic and application based. Typically, education was part of a bundle, and the effects are not easily separable from other components to determine effectiveness. It stands to reason that intubation education is important and should be included. However, a single best delivery method or content could not be established.

Historically, supervised clinical practice was the predominant method to establish proficiency in neonatal intubation.^10^, ^31^ We found among nine studies^12^, ^17^, ^37^, ^38^, ^44^, ^49^, ^53^, ^54^, ^56^ that included supervised clinical practice, in all but one this was preceded by SBT.^49^ Of these studies combining SBT with supervised clinical practice, all but three demonstrated improvements.^12^, ^53^, ^54^ No studies directly compared SBT alone to clinical supervision alone. Overall success in neonatal intubation for novice clinicians improves as exposure to neonatal intubation increases.^10^, ^68^ We suggest that a training bundle using education and SBT is not complete without subsequent structured, supervised clinical practice.

We note previous systematic reviews that found benefit in using video laryngoscopes in both simulation and clinical settings to improve outcomes.^31^, ^69^ We did not identify sufficient new training evidence to justify update systematic reviews on this topic. There is some concern about whether skills acquired using a video laryngoscope might not be transferred or even impair skills when only a direct laryngoscope is subsequently available. However, one study utilising education, SBT and instructor feedback provided evidence of skill transfer between video and direct devices in the simulation setting,^48^ and a further study found that education, SBT and instructor feedback with a video laryngoscope did not impair subsequent clinical outcomes using a direct laryngoscope.^37^ Nevertheless, the transfer of skills from video to direct laryngoscopes or between different video devices deserves further study.

We found little research examining whether benefits of training in neonatal intubation are retained. Literature on resuscitation training suggests that skills including intubation decay after two months^70^, ^71^ with research supporting repeated, short duration refresher training to improve skills.^72^, ^73^, ^74^ Despite this, only one study in this review investigated skill retention at 6–9 months,^39^ suggesting that future studies should examine refresher training and report retention.

### Strengths and Limitations

We limited the review to studies of neonatal intubation training as a standalone intervention, rather than including studies of neonatal resuscitation training. This was to ensure that included studies described reproducible training methods and outcomes pertaining to neonatal intubation itself. We restricted the study to novice clinicians, although a few studies also included participants who were already proficient in intubation. In keeping with usual scoping review methods, we did not conduct any assessment of risk of bias, although in all studies, neither interventions nor outcome assessments were blinded, leading to risk of performance or assessment bias. Our search included trial protocols and conference abstracts as well as published literature but cannot exclude publication bias, in part because the diversity of the included studies precluded *meta*-analysis that might have contributed to detecting it. Nevertheless, we found only one protocol for a trial for which results had not been published in abstract or full-text form, and the review included several studies that reported no improvement in key outcomes.

The review utilised a defined PICOST question, a pre-specified structured protocol, the search strategy was developed with the assistance of an experienced information specialist and rigorous methods of study selection were used.

## Conclusion

In this scoping review we found diverse literature addressing training of novices in neonatal intubation, but we were unable to discern a single ‘standout’ approach. Nevertheless, based on commonality of methods in included studies, we suggest a training bundle that includes education, SBT and subsequent supervised clinical practice for technical skill acquisition. Evidence was extremely limited in relation to skill retention, transfer, and non-technical skills outcomes, suggesting a strong imperative for future research.

## Funding sources

JA is supported by the Mater Foundation through the Betty McGrath education seeding grant.

HL received partial salary support from the Mater Research Institute and Mater Foundation

## CRediT authorship contribution statement

**Jasmine Antoine:** Writing – review & editing, Writing – original draft, Software, Methodology, Formal analysis, Data curation, Conceptualization. **Brian Dunn:** Validation, Formal analysis, Data curation. **Mia McLanders:** Writing – review & editing, Supervision, Conceptualization. **Luke Jardine:** Writing – review & editing, Supervision. **Helen Liley:** Writing – review & editing, Validation, Supervision, Formal analysis, Data curation, Conceptualization.

## Declaration of competing interest

The authors declare the following financial interests/personal relationships which may be considered as potential competing interests: [Professor Helen Liley is an editorial board member for resuscitation plus. The remaining authors have no competing financial interests or personal relationships that could influence the work reported in this paper.].
