# Supplementary material for: Approaches to neonatal intubation training: A scoping review
Source: Resusc Plus. 2024 Sep 23;20:100776. doi: 10.1016/j.resplu.2024.100776 (PMC11456915; doi:10.1016/j.resplu.2024.100776)
Supplement: Supplementary Data 2 [file mmc2.docx]

**Appendix 2: Search Strategy**

The search strategy was developed in conjunction with an information specialist from the University of Queensland, Dr David Honeyman.

The following data bases were searched: PubMed, EMBASE, the Cochrane Central register of Controlled trials (CENTRAL), CINAHL, and Google scholar. The databases were selected as they are a comprehensive coverage of clinical disciplines.

The search strategy was initially run on 1^st^ October 2022 and updated on four occasions, 31^st^ December 2022, 31^st^ October 2023, 13^th^ March 2024, and 21^st^ August 2024. Studies published in English.

**Pubmed:**

**(neonat*[Title/Abstract] OR infant[Title/Abstract] OR newborn[Title/Abstract] OR baby[Title/Abstract] OR babies[Title/Abstract]) AND (intubation [MeSH Terms] OR intubation [Title/Abstract]) AND (train*[Title/Abstract] OR teach*[Title/Abstract] OR educat*[Title/Abstract] OR simulation[Title/Abstract])**

Embase:

(newborn:ab,ti OR infant:ab,ti OR neonat*:ab,ti OR baby:ab,ti) AND (intubation:ab,ti OR 'intubation'/exp) AND (education:ab,ti OR teaching:ab,ti OR simulation:ab,ti)

CENTRAL:

neonat* OR baby OR infant OR newborn in Title Abstract Keyword AND Train* OR Teach* OR Education OR Simulation in Title Abstract Keyword AND intubation in Title Abstract Keyword

CINAHL:

TI (neonatal or neonate or newborn or baby or infant ) OR AB ( neonate or neonatal or newborn or baby or infant) AND ( Training OR Teaching OR Education OR Simulation ) OR AB ( Training OR Teaching OR Education OR Simulation ) AND TI intubation OR AB intubation

Google scholar:

intubation AND Neonate OR neonatal OR infant OR newborn OR baby OR babies AND Train OR teach OR education OR simulation
