# Supplementary material for: Approaches to neonatal intubation training: A scoping review
Source: Resusc Plus. 2024 Sep 23;20:100776. doi: 10.1016/j.resplu.2024.100776 (PMC11456915; doi:10.1016/j.resplu.2024.100776)
Supplement: Supplementary Data 3 [file mmc3.docx]

**Appendix 3: PICOST**

P: Medical practitioners (excluding consultants or attendings), nurses, nurse practitioners (NP) and NP candidates

I: Neonatal intubation training

C: Any other form of neonatal intubation training or no training

O: Technical skills- time to intubation, overall success, first pass success, number of attempts, and complications. Non-technical skills- communication, leadership, and teamwork

S: Randomised, quasi-randomised controlled, cohort, prospective and retrospective trials, qualitative research, and published quality improvement projects

T: Studies published in English up to 21^st^ August 2024
