# Supplementary material for: Approaches to neonatal intubation training: A scoping review
Source: Resusc Plus. 2024 Sep 23;20:100776. doi: 10.1016/j.resplu.2024.100776 (PMC11456915; doi:10.1016/j.resplu.2024.100776)
Supplement: Supplementary Data 4 [file mmc4.docx]

**Appendix 4: Detailed Tables of Included Studies**

| **Table 1: Studies Comparing Training Methods and Measuring Simulation Outcomes** | | | | | | | |
| --- | --- | --- | --- | --- | --- | --- | --- |
| **Author**  **Year**  **Country**  **Setting of Outcome** | **Aim** | **Study Design** | **Participants** | **Intervention** | **Comparator** | **Outcome Measures** | **Results** |
| Al-Wassia  2022  Saudi Arabia  Simulation | To determine the effectiveness of HFM to LFM in improving intubation technical skill and retention | RCT comparing training provided on LFM to HFM. Evaluation using standardised simulated scenario using HFM | 28 paediatric residents | Ed 1 hour + SBT with high-fidelity manikin (HFM): Simbaby® + instructor feedback | Ed 1 hour+ SBT with low-fidelity manikin (LFM): ALS baby + instructor feedback | - Overall success - Time to intubation: start till confirmation mean - Technical skill checklist score | - Overall success: No difference pre and post training - Overall success: No difference between LFM and HFM training. - Time to intubation: No difference pre and post training. LFM 51s and 52s. HFM 46s to 35s - Time to intubation: No difference between LFM and HFM post training - Retention: No difference at 6:9 months in overall success or time to intubation |
| Koele-Schmidt  2016  USA  Simulation | To compare different training models | RCT randomised to one of three training groups. Baseline simulation with no feedback, then education and repeat simulation.  Simulation 3 attempts with DL then VL. | 30 paediatric interns, residents & anaesthetic interns | 1. Ed computer based: neonatal airway anatomy, indication, equipment, technique direct (DL) & video laryngoscope (VL) technique, troubleshooting. Pictures & video + SBT 2. Ed computer based + SBT + standard teaching   Manikin: Sim NewB® | 1. SBT+ standard teaching: didactic ed+ demo with focus on equipment, DL intubation technique, ETT position, VL technique | - Time to intubation (not defined) - Confidence in intubation | - Time to intubation: Significant improvement in time to successful intubation post all teaching group in both DL 22.0s vs 14.7 s p=0.002 and VL 42.2 s vs 26.8 s p=0.003 - Time to intubation: No difference identified between training interventions |

| **Table 2: Studies Comparing Training Methods and Measuring Clinical Outcomes** | | | | | | | |
| --- | --- | --- | --- | --- | --- | --- | --- |
| **Author**  **Year**  **Country**  **Setting of Outcome** | **Aim** | **Study Design** | **Participants** | **Intervention** | **Comparator** | **Outcome Measures** | **Results** |
| Akierman 2002  Canada  DR | To compare intubation training programs across three sites | Retrospective observational study reviewing training programs at three sites | 52 residents, fellows, paediatrician, neonatologist, nurses & respiratory therapist | Training program: Ed + SBT with part task trainer or cat lab + instructor feedback + supervised clinical practice | Nil | - Overall success: doesn’t define - Number of intubations per attended delivery | - Overall success: No significant difference in rate across three sites |
| Finan  2012  Canada NICU | Compare sim performance pre to post training & success compared to historical cohort | Prospective observational study. Post training testing in sim + clinical intubations performed during the rotation. Performance was compared with historical dataset | 13 first year paediatric residents | Training program: Ed + demo + SBT + instructor feedback: 2 hours  High-fidelity infant manikin | Historic cohort: no additional training | In sim:   - Checklist - Global rating score   In clinical:   - Overall success: within 2 attempts - Time to intubation - Checklist - Global rating scale | - Overall success: 67.5% post training compared to 63.15% historic p=0.06 - Time to intubation: No difference 71.23s to cohort 59.94s p=0.24 - Global rating and checklist scores: improved post training - Global rating and checklist scores: in the clinical setting, scores did not differ from baseline |
| Gizicki  2023  Canada  NICU | To assess if just in time training (JTT) is superior to video education for intubation training | RCT. Post training supervised clinical intubation for outcome | 112 paediatric residents | JTT: Ed with VL/DL+ SBT+ instructor feedback: 10 minutes  Manikin: Newborn Anne® | Video education with VL/DL: 5minutes | - FPS - Overall success: ≤3 attempts - Number of attempts - Time to intubation: blade in mouth to out of mouth - Complications | - FPS: Significantly higher in JTT group 54% vs 41% - Overall success: No difference - Number of attempts: median number significantly lower in JTT - Time to intubation: mean significantly faster in JTT group 35s compared with video training 62s - Complications: significantly more mucosal trauma in JTT group, no other differences in complication - Use of VL: video training group used more frequently may account longer time |
| O'Shea 2021  UK  DR & NICU | To assess the first attempt intubation success following training program | Prospective observational study comparing intervention epoch to historical data. Outcome measured at supervised clinical intubations | 102 residents, registrars, fellows and NNPs | Training program:  Ed+ SBT with DL/VL+ instructor feedback + pre-procedure pause + introduction of VL+ supervised clinical intubations  Manikin not specified | Historic cohort | - FPS - Overall success - Number of attempts | - First pass success: junior operators 61% post training vs 40% historic - Overall success: junior operators improved varied between 58-64% vs. historic 35%-48% - Success rates were higher using VL for clinicians at all levels of training |
| Rumpel  2022  USA  NICU | To determine if intervention training participants have higher success in clinical DL intubation the following month | Quasi: RCT different training programs. Outcome measured clinical intubation using DL | 102 paediatric residents | Intervention training program:30-minute Ed: equipment, ETT size, depth, steps + SBT using Premature Anne ® manikin with VL used directly+ one: one instructor feedback + repeat SBT intubation time <15s + standard training | Standard training: 15min SBT on term part task manikin with DL+ instructor feedback + supervised clinical practice | - FPS - Overall success - Total number of patient intubations (median) | - FPS: No difference between training groups 35% intervention vs 28% standard p=0.256. - Overall success : No difference between training groups 50% intervention vs. 55% control, p=0.215. |

| **Table 3: Training with Video Laryngoscopy Compared to Direct Laryngoscopy Measured in Simulation** | | | | | | | | | | | |
| --- | --- | --- | --- | --- | --- | --- | --- | --- | --- | --- | --- |
| **Author**  **Year**  **Country**  **Setting of Outcome** | **Aim** | **Study Design** | **Participants** | **Intervention** | | **Comparator** | | **Training Method** | **Outcome Measures** | **Results** | |
| Grgurich  2016  USA  Simulation | To determine if VL improved intubation | RCT: crossover. Post training participants intubated a manikin twice, once with VL and once with DL | 19 nurses | CMAC® video laryngoscope  with video output screen visible (VL) | | CMAC® video laryngoscope used directly with video output screen not visible (DL) | | Ed 5 min: anatomy, indication, equipment, complication, difficult airway management, troubleshooting & SBT with DL/VL  Manikin: Neonatal intubation trainer | - Time to intubation: blade in mouth until first breath delivered - Number of attempts - Percentage of glottic opening score (POGO) - Time to first view: blade in mouth until view - Time to best POGO | - Time intubation: No difference - Number of attempts: No difference | |
| Johnston  2014  USA  Simulation | To evaluate the efficacy of exploration based learning on performance with VL | RCT: crossover. Post training participants intubated with VL and DL on newborn manikin and 3:month infant manikin | 64 participants: 40 paediatric residents, 5 fellows , 10 faculty PICU, NICU & ED.  9 anaesthetics | Glidescope® video laryngoscope (VL) | | DL | | Ed: video on VL + SBT VL/DL: 1hour  Manikin: Laerdal Infant part task trainer (3month) | - Number of attempts: blade in mouth - Time to intubation: blade in mouth until chest rise - Intubation >5min - Participant confidence | - Number attempts: no difference - Time to intubation: faster with DL 26.3s to VL 46.5s p <0.001 - Time to intubation residents: faster with DL 27.8s to VL 54.5s p <0.001. - Time to intubation fellow: no difference DL 21.4s to VL 26s - No difference in failed intubations | |
| Komasawa  2015  Japan  Simulation | To compare the accuracy and ease of intubation with DL and VL during chest compressions | RCT: crossover. Participants 4 simulation 1. DL chest compression, 2. DL no compression, 3. VL chest compression, 4. VL no chest compression | 23 anaesthetic trainees with no infant airway experience | Pentax ® VL with and without chest compressions | | DL with and without chest compressions | | 10min SBT DL/VL+ instructor feedback  Manikin: Newborn Anne® (term) | - Overall success - Time to intubation - Participant rating on ease of use | - Overall success : No difference in DL and VL without chest compressions. - Overall success: Significantly higher VL compared to DL during chest compressions - Time to intubation : significantly shorter with VL than DL in both chest and no chest compressions - Ease of use: VL was significantly easier to use than DL | |
| Musharaf  2020  Canada  Simulation | To compare the success rate of DL & VL | Non-randomised cross over. Participants divided into three groups based on frequency of intubation. Post training participants intubated with DL and VL in a non-random sequence on term manikin. | 26 participants: 8 residents, 9 transport nurses & respiratory therapist, 9 neonatal nurse practitioners and physician | Glidescope® VL | | DL | | Ed: video VL+ demonstration DL/VL  Manikin: Sim NewB® | - FPS - Number of attempts - Time to intubation: blade in mouth to out - Participant device preference | - FPS: No difference DL and VL for residents, transport nurses and respiratory therapist - FPS: Physicians and nurse practitioners DL 100% than VL 88.9% - Time intubation: No difference in the median time for residents, nurses and respiratory therapist - Time intubation: Physician and nurse practitioners were VL 11s to DL 15s respectively p0.024 - Preference: Residents preferred VL to DL - Preference: No preference was seen in the other groups | |
| Nair  2017  USA  Simulation | To determine whether VL training compared to DL improves neonatal intubation | RCT: Block randomised. Following training testing on Sim NewB using DL. | 123 participants: 64 paediatric residents or neonatology fellows, 59 respiratory therapy students <5 successful intubations | CMAC® VL with training in VL & DL | | DL  with training in DL only | | Ed PowerPoint + SBT 15min + instructor feedback with allocated device  Manikin: Newborn part task trainer | - FPS - Number of attempts - Time to intubation: blade in mouth to passage ETT trachea | | - FPS: no difference, 69% DL vs 61% VL/DL trained p=0.35 - Number of attempts: no difference between group - Time to intubation: no difference, 25s DL vs 26.5s VL/DL trained p=0.27 |
| Parmekar  2017  USA  Simulation | To compare DL & VL on intubation success | RCT: cross over. Post training participants intubated first pass then 3 resuscitation simulations with device trained in. Then crossover and intubate first pass then 3 resuscitation simulation with second device. | 100 paediatric residents | CMAC® VL | | | DL | Ed in allocated device 20min + SBT with allocated device 30 min + instructor feedback  Manikin: Sim NewB® | - FPS - Overall success - Number of attempts - Time to intubation: blade in to blade out on successful attempt   Self-reported confidence | | - FPS: VL trained group with VL 88% versus DL trained group with DL 63% p=0.008 - FPS: VL trained group with VL 93% versus DL trained group with VL 74% p=0.034 on second scenario. No difference other VL scenario - Overall success: VL trained group no difference: VL 88% to DL 93% - Overall success: DL trained group: significantly increased success DL 63% to VL 89% p=0.008 - Number attempts: No difference seen VL or DL post cross over - Time intubation: No significant difference between VL and DL group on first attempt - Time to intubation: VL trained group got significantly faster with DL, VL 29s to DL 17s respectively p<0.001 - Time to intubation: DL trained group no difference using DL to VL - Self-reported confidence significantly improved post training intervention |
| Shaylor  Israel  2023  Simulation | To investigate the success rate of intubation with VL to DL with manikin | RCT: cross over study. Post training intubate manikin. Random VL and DL order. | 23 Paediatric residents | CMAC® VL + McGrath VL | | DL | | Ed video DL/both VL 5 min+ SBT DL/both VL 15min+ Instructor feedback  Manikin: Pierre-Robin (PRS) model 0:6 months | - Overall success: within 3 attempts - Time to intubation: blade in mouth till ETT through vocal cords - Anaesthetic review of success and grade view | | - Overall success: significantly higher DL 74% than VL-CMAC VL 22%, McGrath VL 30% p=0.04 - Overall success: no difference between VL - Time to intubation: significantly shorter DL 18s than VL-CMAC VL 56s and McGrath VL 29s p=0.01 |
| Zhou  2020  China  Simulation | To compare the difference between DL and VL in intubation | Prospective Observational simulation study. Post training 3 testing performed  1. 47 trainee: 25 experienced staff & 22 less experienced staff Test in sim either VL or DL with instructor feedback  2. 23 trainee:12 experienced staff & 11 less experienced staff Test in sim with chest compressions either VL or DL with instructor feedback  3. 52 midwife (novice)  Further training demo of VL or DL intubation + test in sim with instructor feedback | 99 participants: 62 nurses & midwives, 37 doctors* | | VDO:100 c ® VL | | DL | Skill station with demo + SBT + instructor feedback  3 days  Manikin: Sim NewB | - Overall success - Time to intubation: Blade in blade out, mean | | - Overall success: for experienced staff significantly lower in VL 48% vs DL 88% - Overall success: For less experienced staff no significant difference in VL & DL - Overall success: Novice staff post education success significantly higher in VL 96% vs DL 69% - Time to intubation: significantly longer for experienced staff using VL 24.1s vs DL 18.1 - Time to intubation: Less experienced staff no difference between VL and DL - Time to intubation: Novice staff post education intubation time significantly shorter in VL 19.6s vs DL 28s - Time to intubation: No difference in time between experienced and less experienced staff |

| **Table 4: Training with Video Laryngoscopy Compared to Direct Laryngoscopy and Measuring Clinical Outcomes** | | | | | | | | |
| --- | --- | --- | --- | --- | --- | --- | --- | --- |
| **Author**  **Year**  **Country**  **Setting of Outcome** | **Aim** | **Study Design** | **Participants** | **Intervention** | **Comparator** | **Training Method** | **Outcome Measures** | **Results** |
| Abid  2021  USA  Retrieval | To examine success post VL introduction in retrieval team over 8 years | Retrospective observational study over 8 years | 135 participants: 123 critical care RN & 12 paramedics | CMAC® VL | DL | Ed + SBT + instructor feedback + supervised clinical practice: 1 month  All DL/VL  Manikin: Airway trainer | - FPS - Number of attempts: blade into mouth | - FPS: No difference between DL &VL - VL use increased over the study - Number of attempts: neonatal data not reported separately |
| Coutu  2022  Canada  Retrieval | To improve first pass intubation success with introduction of VL | Observational study post implementation of VL and training. | 103 participants: 67 RN, 30 respiratory therapist, 6 doctors- fellows and physicians | CMAC® VL & training | DL: Historic cohort | VL Ed + SBT + debrief clinical recorded intubations  Manikin: Part task trainer | - FPS - Overall success - Number of attempts - Complications | - FPS: No difference post introduction of VL & training program - Overall success: Improved 89% pre to 99% post p=0.002 - Number of attempts: No difference - Complications: 2% cases, no comparison to pre VL & training |
| Moussa  2016  Canada  NICU | To assess if VL is superior to DL in acquiring skills in neonatal intubation. To determine if skill in VL are transferrable to DL | RCT: cross over in 2 phases.  1. Participants were randomised to initially intubate with either VL or DL for at least 3 successful intubations (max 7 patients)  2. Transfer test all intubations performed with DL | 34 first year paediatric trainees | CMAC® VL | DL | DL Ed + DL SBT+ instructor feedback + supervised clinical practice  VL group: had additional Ed in VL  Manikin: Neonatal intubation trainer Laerdal® | - Overall success: less than 4 attempts - Number of attempts - Time to intubation: blade in mouth to blade out, median - Complications | Phase 1   - Overall success: Higher VL 75.2% vs DL 63.4% p=0.03 - Number of attempts: No difference - Time to intubation: Longer in VL 57s vs DL 45s p=0.08 - Complications: Significantly more mucosal trauma with DL vs VL   Phase 2:   - Overall success: No difference between phases VL group phase 1 75% phase 2 with DL 63% - Overall success: No difference between phases DL group phase 1 DL 63% phase 2 DL 77% |
| Saran  2019  India  OT | To determine the efficacy and safety of VL guided verbal feedback compared to DL verbal feedback | RCT: cross over, elective intubations of infants in OT. Each trainee performed 3 intubation with device and switched to device to perform 3 more intubations | 24 anaesthetic trainees | CMAC® VL + supervised clinical practice | CMAC VL used directly + supervised clinical practice | Ed: video of intubation with DL& VL + supervised clinical practice during testing | - FPS - Time to intubation: blade in mouth to end tidal co2 - Complications - Time to best view | - FPS: significant higher in VL 83.3% vs DL 44.4% p<0.001 - Time to intubation: 30 s for VL vs 41.7 s for DL P < 0.001 - Time best view: 19.8 s VL vs 26.8 s for DL P < 0.001 - Complications: fewer oesophageal intubations and desaturations |
| Yankowski2022  USA  NICU | To determine the impact of a VL and DL/VL curriculum on intubation | RCT. Randomised to VL and DL teaching or DL only teaching. Survey prior and after session. Tested in sim then clinical environment. | 49 participants: 47 paediatric residents, 2 advance practice clinicians | DL/VL training | DL training | Ed + SBT in allocated laryngoscope+ instructor feedback  Manikin: not specified | In sim environment:   - FPS - Number of attempts - Time to intubation - Learner confidence   In clinical environment:   - Overall success | In sim environment:   - FPS: no difference - Number attempts: not reported - Time to intubation: no difference - No difference in learner evaluation of curriculum   In clinical setting:   - Overall success: higher in DL 70% vs VL/DL group 33% p=0.048 - Overall success for residents: no difference |

| **Table 5 : Studies Comparing Training with Video Laryngoscopes used Directly with Supervision to Direct Laryngoscopy with Supervision (Clinical Outcomes)** | | | | | | | | |
| --- | --- | --- | --- | --- | --- | --- | --- | --- |
| **Author**  **Year**  **Country**  **Setting of Outcome** | **Aim** | **Study Design** | **Participants** | **Intervention** | **Comparator** | **Training Method** | **Outcome Measures** | **Results** |
| O'Shea  2015  Australia  DR & NICU | To determine if supervision with VL output improves participant’s first attempt success | RCT, stratified by use of premedication | 36 paediatric residents < 6 months tertiary neonatal experience | VL used directly with supervisor able to see airway via VL output: Acutronic® (VL sup) | VL used directly with supervisor unable to see VL output (VL covered) | Ed + SBT+ supervised clinical practice + debrief clinical intubations | - FPS - Time to intubation: blade in to blade out - Complications | - FPS: VL sup 66% vs VL covered 41%, when no premedication given no difference - Time to intubate: No difference duration of attempts - Complication: No difference in rate of hypoxia, bradycardia |
| Volz  2018  USA  Non emergent NICU | To evaluate the intubation success rates of residents who used VL sup vs DL | RCT | 48 residents | VL used directly with supervisor able to see airway via VL output :CMAC (VL sup) | DL: Rusch® laryngoscope | SDL video + Ed+ SBT + supervised clinical practice  Part task trainer | - FPS - Overall success ≤ 2 attempts - Time to intubation - Complications | - FPS: VL sup 50% vs 17% DL p<0.05 - Overall success : VL sup 57% vs DL 33% p<0.02 - Time to intubate: No difference - Complications: No difference |

| **Table 6. Virtual Reality Assisted Training with Simulation Outcomes** | | | | | | | | |
| --- | --- | --- | --- | --- | --- | --- | --- | --- |
| **Author**  **Year**  **Country** | **Aim** | **Study Design** | **Participants** | **Intervention** | **Comparator** | **Training Method** | **Outcome Measures** | **Results** |
| Dias  2021  USA | To investigate whether ARVL improved novice intubation proficiency vs. VL or DL | RCT. Participants randomly assigned to ARVL, VL or DL for 5 x attempts at intubation of term manikin with feedback | 45 NICU nurses | 1. Augmented reality assisted VL (ARVL)  2. VL | DL | Ed+ instructor feedback during testing + debrief  Manikin: Life/form Basic Infant CRiSis® | - Overall success - Time to intubation - Time to visualise airway - Time between airway identification and intubation | - Overall success: Significant improvement ARVL and VL vs DL p<0.001 - Time to intubation: Median time to intubation faster in ARVL and VL vs. DL 21.6s, 20.7s & 35.6s, respectively, p<0.001 |
| O’Sullivan  2022  USA | To investigate whether VR is non-inferior to demonstration for neonatal intubation education | RCT. Participants randomly assigned to VR or demonstration before intubating manikin | 23 clinicians | 360 degree immersive virtual reality training video (VR) | In-person skill demonstration | 20min Ed allocated video or demo  Manikin: Premature Anne® | - Overall success - Number of attempts - Time to intubation: median time - Knowledge score - Skills score | - Overall success: No difference between groups - Time to intubation: No difference VR 60s vs demonstration 57s - Skill score: No significant difference - Participants randomised to VR, 35% complained of motion sickness |

| **Table 7: Cognitive Aids with Outcomes Measured in Simulation** | | | | | | | | |
| --- | --- | --- | --- | --- | --- | --- | --- | --- |
| **Author**  **Year**  **Country** | **Aim** | **Study Design** | **Participants** | **Intervention** | **Comparator** | **Training Method** | **Outcome Measures** | **Results** |
| Hawkes  2013  Ireland | To determine whether app improved trainee knowledge and intubation performance | Prospective observational study. Participants completed knowledge questionnaire and were assessed intubating term manikin. Post training the questionnaire and simulation repeated | 20 participants: 12 paediatric fellows, 8 residents | NeoTube application education | Pre application | Ed: 15:minute neonatal intubation instruction application with videos NeoTube  Manikin: Term newborn | - Time to intubation - Knowledge score - Skill score | - Time to intubation: Pre 39s significantly reduced post training to 31s p0.044 - Improved knowledge scores p<0.001 |

| **Table 8: Cognitive Aid with Clinical Outcomes** | | | | | | | | |
| --- | --- | --- | --- | --- | --- | --- | --- | --- |
| **Author**  **Year**  **Country**  **Setting of Outcome** | **Aim** | **Study Design** | **Participants** | **Intervention** | **Comparator** | **Training Method** | **Outcome Measures** | **Results** |
| Dalrymple  2022  Australia  Retrieval | To determine whether first-pass intubation success rates improve | Prospective observational cohort study. | 16 neonatal or anaesthetic trainees | Daily low-fidelity simulation with checklist | Historic cohort | Daily  SBT + supervised clinical practice 1 day per term in OT  Neonatal manikin | - FPS - Time to intubation: mean - Complications - Intubation view grading | - FPS: significantly improved 78 vs. historic 59% p=0.032 - Complications: nil severe recorded - Time to intubation: 27s for successful, no comparator data - Poor compliance with simulation 43.5% |

**Footnotes to all tables:**

Abbreviations: DR; delivery room, NICU; neonatal intensive care unit, OT; operating theatre, Sim; simulation, Clin; clinical,VL:video laryngoscope, DL; direct laryngoscope, SDL; self-directed learning, Ed; education, SBT; simulation-based training, Demo; demonstration, RCT; randomised controlled trial, qRCT; quasi randomised controlled trial, FPS: first pass success

* Doctors: includes junior and senior medical officers, RT; respiratory therapist, RN; registered nurse, NNP; neonatal nurse practitioners
