# Supplementary material for: Approaches to neonatal intubation training: A scoping review
Source: Resusc Plus. 2024 Sep 23;20:100776. doi: 10.1016/j.resplu.2024.100776 (PMC11456915; doi:10.1016/j.resplu.2024.100776)
Supplement: Supplementary Data 5 [file mmc5.docx]

**Appendix 5: Excluded Studies at Full Text Review**

| **Reason** | **Number** | **References** |
| --- | --- | --- |
| Training methods were not described or Wrong training (training described neonatal resuscitation program with insufficient details in relation to intubation component) | 18 | Bayoumi ^1^, Bhatia, Stewart ^2^, Bismilla, Finan ^3^, Brady, Kovatis ^4^, Chan, Mistry ^5^, DeMeo, Katakam ^6^, Dongara, Modi ^7^, Donoghue, Nishisaki ^8^, Donoghue, Ades ^9^, Hadfield, Sawyer ^10^, Haubner, Barry ^11^, Johnston, Sawyer ^12^, Moussa, Luangxay ^13^, O'Donnell, Kamlin ^14^, Salis-Soglio, Hummler ^15^, Singh, Santosh ^16^, Soghier, Walsh ^17^, Ferris, Corrigan ^18^ |
| Wrong participant population (including medical students, nursing students, only consultant doctors) | 7 | Assaad, Lachance ^19^, Covelli, Bardelli ^20^, Dvorsky, Rings ^21^, Ernst, Cline ^22^, Iacovidou, Bassiakou ^23^, Lejus-Bourdeau, Pousset ^24^, Song, Choi ^25^ |
| Wrong study design | 12 | Andreatta, Dooley-Hash ^26^, Andreatta, Klotz ^27^, Campbell and Finan ^28^, Downes, Narendran ^29^, Evans, Shults ^30^, Falck, Escobedo ^31^, Finer and Rich ^32^, Gaies, Landrigan ^33^, Gariépy-Assal, Janaillac ^34^, Leone ^35^, Leone, Rich ^36^, Ryan, Clark ^37^ |
| Wrong outcome measures | 5 | Benfield, Flaksman ^38^, Chitkara, Bennett ^39^, Hulmes and El-Kafrawy ^40^, Jackson, Sinnott ^41^, O'Shea, Kirolos ^42^ |
| Wrong patient population (including not newborn or neonates or first admission or manikins over 3 months) | 4 | Fonte, Oulego-Erroz ^43^, Haug and Srivastava ^44^, Miller, Monuteaux ^45^, Sakurai and Tamura ^46^ |
| Duplicate data or duplicate study | 4 | Al-Wassia, Bamehriz ^47^, Donoghue, Ades ^9^, Gariépy-Assal, Janaillac ^34^, Saran, Dave ^48^ |
| Duplicate data: abstract that had subsequent publication | 4 | Campbell and O'Shea ^49^, Moussa, Luangxay ^50^, Edwards, Godde ^51^, Parmekar, Lingappan ^52^ |
| Full text published in language other than English | 1 | Lenclen, Narcy ^53^ |
| Clinical trial registration with no associated publication | 1 | Nct ^54^ |
| Abstract only with insufficient details | 1 | Wong, Elgin ^55^ |
| Total | 57 |  |

**Reference List**

1. Bayoumi M. Endotracheal intubation in inter-professional neonatal emergencies simulation workshops versus neonatal resuscitation program courses Journal of Community and Public Health Nursing. 2018;4:15.

2. Bhatia M, Stewart AE, Wallace A, Kumar A, Malhotra A. Evaluation of an In-Situ Neonatal Resuscitation Simulation Program Using the New World Kirkpatrick Model. Clinical Simulation in Nursing. 2021;50:27-37.

3. Bismilla Z, Finan E, McNamara PJ, LeBlanc V, Jefferies A, Whyte H. Failure of pediatric and neonatal trainees to meet Canadian Neonatal Resuscitation Program standards for neonatal intubation. J Perinatol. 2010;30:182-7.

4. Brady J, Kovatis K, CL OAD, Gray M, Ades A. What Do NICU Fellows Identify as Important for Achieving Competency in Neonatal Intubation? Neonatology. 2019;116:10-6.

5. Chan NH, Mistry N, Campbell DM. A Simulation-Based Pilot Study of a Mobile Application (NRP Prompt) as a Cognitive Aid for Neonatal Resuscitation Training. Simul Healthc. 2019;14:146-56.

6. DeMeo SD, Katakam L, Goldberg RN, Tanaka D. Predicting neonatal intubation competency in trainees. Pediatrics. 2015;135:e1229-36.

7. Dongara AR, Modi JJ, Nimbalkar SM, Desai RG. Proficiency of residents and fellows in performing neonatal intubation. Indian Pediatr. 2014;51:561-4.

8. Donoghue A, Nishisaki A, Ades A, Nadkarni V. Videolaryngscopy versus standard direct laryngoscopy in pediatric intubation: a simulation study. Critical Care Medicine. 2009;37.

9. Donoghue AJ, Ades AM, Nishisaki A, Deutsch ES. Videolaryngoscopy versus direct laryngoscopy in simulated pediatric intubation. Ann Emerg Med. 2013;61:271-7.

10. Hadfield BR, Sawyer T, Moreira AG, Farner R, Vasquez MM. Rapid cycle deliberate practice improves resident performance during ELBW resuscitation. J Neonatal Perinatal Med. 2024;17:31-40.

11. Haubner LY, Barry JS, Johnston LC, Soghier L, Tatum PM, Kessler D, et al. Neonatal intubation performance: room for improvement in tertiary neonatal intensive care units. Resuscitation. 2013;84:1359-64.

12. Johnston L, Sawyer T, Ades A, Moussa A, Zenge J, Jung P, et al. Impact of Physician Training Level on Neonatal Tracheal Intubation Success Rates and Adverse Events: A Report from National Emergency Airway Registry for Neonates (NEAR4NEOS). Neonatology. 2021;118:434-42.

13. Moussa A, Luangxay Y, Tremblay S, Lavoie J, Aube G, Savoie E, et al. Neonatal endotracheal intubation learned with videolaryngoscope is maintained with classic laryngoscope: phase 2 of a crossover randomized trial. Paediatrics and child health (Canada). 2015;20:e33.

14. O'Donnell CP, Kamlin CO, Davis PG, Morley CJ. Endotracheal intubation attempts during neonatal resuscitation: success rates, duration, and adverse effects. Pediatrics. 2006;117:e16-21.

15. Salis-Soglio N, Hummler H, Schwarz S, Mendler MR. Success rate and duration of orotracheal intubation of premature infants by healthcare providers with different levels of experience using a video laryngoscope as compared to direct laryngoscopy in a simulation-based setting. Front Pediatr. 2022;10:1031847-.

16. Singh J, Santosh S, Wyllie JP, Mellon A. Effects of a course in neonatal resuscitation-evaluation of an educational intervention on the standard of neonatal resuscitation. Resuscitation. 2006;68:385-9.

17. Soghier LM, Walsh HA, Goldman EF, Fratantoni KR. Simulation for neonatal endotracheal intubation training: How different is it from clinical practice? Simul Healthc. 2022;17:e83-e90.

18. Ferris KCA, Corrigan N, Armstrong D, Hughes A, Thiraviaraj A. Intubation in the neonatal unit-improving our performance and fostering human factor awareness. Archives of Disease in Childhood. 2019;104:A167-A8.

19. Assaad MA, Lachance C, Moussa A. Learning Neonatal Intubation Using the Videolaryngoscope: A Randomized Trial on Mannequins. Simul Healthc. 2016;11:190-3.

20. Covelli A, Bardelli S, Scaramuzzo RT, Sigali E, Ciantelli M, Del Pistoia M, et al. Effectiveness of a new sensorized videolaryngoscope for retraining on neonatal intubation in simulation environment. Ital J Pediatr. 2020;46:13.

21. Dvorsky R, Rings F, Bibl K, Roessler L, Kumer L, Steinbauer P, et al. Real-Time Intubation and Ventilation Feedback: a Randomized Controlled Simulation Study. Pediatrics. 2023;151.

22. Ernst KD, Cline WL, Dannaway DC, Davis EM, Anderson MP, Atchley CB, et al. Weekly and consecutive day neonatal intubation training: comparable on a pediatrics clerkship. Acad Med. 2014;89:505-10.

23. Iacovidou N, Bassiakou E, Stroumpoulis K, Koudouna E, Aroni F, Papalois A, et al. Conventional direct laryngoscopy versus videolaryngoscopy with the GlideScope®: a neonatal manikin study with inexperienced intubators. Am J Perinatol. 2011;28:201-6.

24. Lejus-Bourdeau C, Pousset F, Magne C, Bazin O, Grillot N, Pichenot V. Low-cost versus high-fidelity pediatric simulators for difficult airway management training: a randomized study in continuing medical education. Braz J Anesthesiol. 2023;73:250-7.

25. Song CH, Choi A, Roebuck B, Dannaway D, Anderson MP. Real-time, Media-enhanced Feedback Improves Neonatal Intubation Skills. Pediatrics. 2018;142:234-.

26. Andreatta PB, Dooley-Hash SL, Klotz JJ, Hauptman JG, Bea Biddinger DVM, House JB. Retention curves for pediatric and neonatal intubation skills after simulation based training. Pediatr Emerg Care. 2016;32:71-6.

27. Andreatta PB, Klotz JJ, Dooley-Hash SL, Hauptman JG, Biddinger B, House JB. Performance-based comparison of neonatal intubation training outcomes: simulator and live animal. Adv Neonatal Care. 2015;15:56-64.

28. Campbell DM, Finan E. Impact of video-debriefing following simulated neonatal reuscitation in inter-professional teams. Paediatr Child Health. 2014;19.

29. Downes KJ, Narendran V, Meinzen-Derr J, McClanahan S, Akinbi HT. The lost art of intubation: assessing opportunities for residents to perform neonatal intubation. J Perinatol. 2012;32:927-32.

30. Evans P, Shults J, Weinberg DD, Napolitano N, Ades A, Johnston L, et al. Intubation competence during neonatal fellowship training. Pediatrics. 2021;148.

31. Falck AJ, Escobedo MB, Baillargeon JG, Villard LG, Gunkel JH. Proficiency of pediatric residents in performing neonatal endotracheal intubation. Pediatrics. 2003;112:1242-7.

32. Finer N, Rich W. Neonatal resuscitation for the preterm infant: evidence versus practice. J Perinatol. 2010;30 Suppl:S57-66.

33. Gaies MG, Landrigan CP, Hafler JP, Sandora TJ. Assessing procedural skills training in pediatric residency programs. Pediatrics. 2007;120:715-22.

34. Gariépy-Assal L, Janaillac M, Ethier G, Pennaforte T, Lachance C, Barrington KJ, et al. A tiny baby intubation team improves endotracheal intubation success rate but decreases residents' training opportunities. J Perinatol. 2023;43:215-9.

35. Leone TA. Procedural training in neonatology. Curr Opin Pediatr. 2023;35:204-8.

36. Leone TA, Rich W, Finer NN. Neonatal intubation: success of pediatric trainees. J Pediatr. 2005;146:638-41.

37. Ryan CA, Clark LM, Malone A, Ahmed S. The effect of a structured neonatal resuscitation program on delivery room practices. Neonatal Netw. 1999;18:25-30.

38. Benfield DG, Flaksman RJ, Lin TH, Kantak AD, Kokomoor FW, Vollman JH. Teaching intubation skills using newly deceased infants. Jama. 1991;265:2360-3.

39. Chitkara R, Bennett M, Bohnert J, Yamada N, Fuerch J, Halamek LP, et al. In Situ Simulation and Clinical Outcomes in Infants Born Preterm. J Pediatr. 2023;263:113715.

40. Hulmes E, El-Kafrawy U. Enhancing trainee endotracheal intubation skills on the NICU. Infant. 2019;15:238-9.

41. Jackson S, Sinnott M, Johnston J. 1024 Simulation training for video laryngoscopy for neonatal intubation. Archives of Disease in Childhood. 2021;106:A196.

42. O'Shea JE, Kirolos S, Thio M, Kamlin COF, Davis PG. Neonatal videolaryngoscopy as a teaching aid: the trainees' perspective. Arch Dis Child Fetal Neonatal Ed. 2021;106:168-71.

43. Fonte M, Oulego-Erroz I, Nadkarni L, Sánchez-Santos L, Iglesias-Vásquez A, Rodríguez-Núñez A. A randomized comparison of the GlideScope videolaryngoscope to the standard laryngoscopy for intubation by pediatric residents in simulated easy and difficult infant airway scenarios. Pediatr Emerg Care. 2011;27:398-402.

44. Haug JL, Srivastava G. Videolaryngoscopy as an educational tool for the novice pediatric intubator: a comparative study. Academic emergency medicine. 2012;19:S189.

45. Miller KA, Monuteaux MC, Aftab S, Lynn A, Hillier D, Nagler J. A Randomized Controlled Trial of a Video-Enhanced Advanced Airway Curriculum for Pediatric Residents. Acad Med. 2018;93:1858-64.

46. Sakurai Y, Tamura M. Efficacy of the Airway Scope (Pentax-AWS) for training in pediatric intubation. Pediatr Int. 2015;57:217-21.

47. Al-Wassia H, Bamehriz M, Atta G, Saltah H, Arab A, Boker A. Effect of training using high-versus low-fidelity simulator mannequins on neonatal intubation skills of pediatric residents: a randomized controlled trial. BMC Med Educ. 2022;22:497.

48. Saran A, Dave NM, Karnik PP. Efficacy and safety of videolaryngoscopy-guided verbal feedback to teach neonatal and infant intubation. A prospective randomised cross over study. Indian J Anaesth. 2019;63:791-6.

49. Campbell M, O'Shea J. Neonatal videolaryngoscopy as a teaching aid-the trainees' perspective from an RCT. Scottish medical journal. 2015;60:NP6.

50. Moussa A, Luangxay Y, Tremblay S, Lavoie J, Aube G, Savoie E, et al. Videolaryngoscope vs classic laryngoscope in teaching neonatal endotracheal intubation: a randomized controlled trial. Paediatrics and child health (canada). 2014;19:e50.

51. Edwards G, Godde C, Brunton A, O'Shea J. Improving neonatal intubation success and safety in a large Scottish tertiary neonatal unit. Scottish Medical Journal. 2022;67:62-3.

52. Parmekar S, Lingappan K, Arnold JL, Tyner C, Pammi M. Videolaryngoscopy versus direct laryngoscopy for neonatal endotracheal intubation by pediatric trainees: a simulation study. Pediatrics. 2018;141.

53. Lenclen R, Narcy P, Castela F, Huard F. Evaluation of an educational intervention on the standard of neonatal resuscitation: orotracheal versus nasotracheal intubation. Arch Pediatr. 2009;16:337-42.

54. Nct. Resident Training Enhanced by New Innovations: teleintubation. <https://clinicaltrialsgov/show/NCT02572427>. 2015.

55. Wong S, Elgin T, Spellman E. Utilizing an Intensive Training Program to Improve Neonatal-pediatric Transport Intubation Success. Pediatrics. 2022;149:960.
