# Supplementary material for: Approaches to neonatal intubation training: A scoping review
Source: Resusc Plus. 2024 Sep 23;20:100776. doi: 10.1016/j.resplu.2024.100776 (PMC11456915; doi:10.1016/j.resplu.2024.100776)
Supplement: Supplementary Data 6 [file mmc6.docx]

**Appendix 6: Study Location Map**

**1** UK

**1** Ireland

**6** Canada

**11** USA

**1** India

**1** Japan

**1** China

**1** Saudi Arabia

**1** Israel

**2** Australia
