# Supplementary material for: Approaches to neonatal intubation training: A scoping review
Source: Resusc Plus. 2024 Sep 23;20:100776. doi: 10.1016/j.resplu.2024.100776 (PMC11456915; doi:10.1016/j.resplu.2024.100776)

**Appendix 7 : Frequency of Clinician Participants**


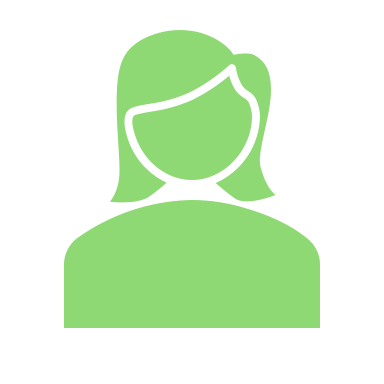


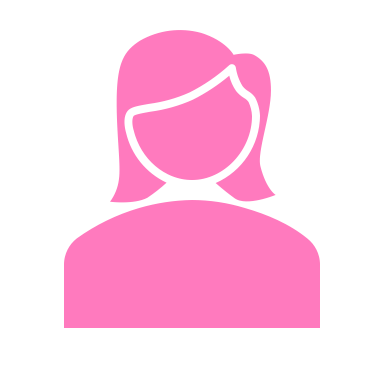

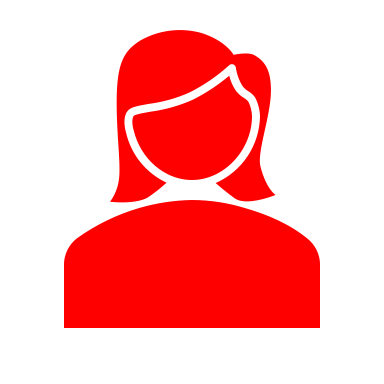

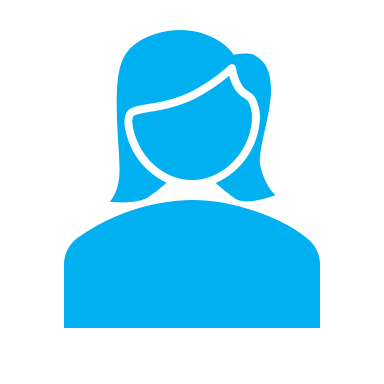

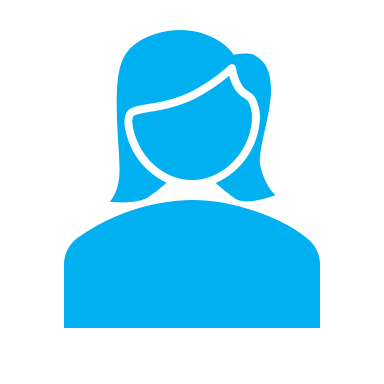

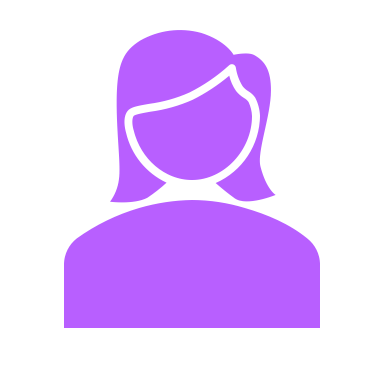

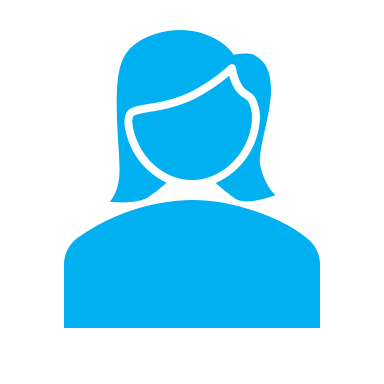

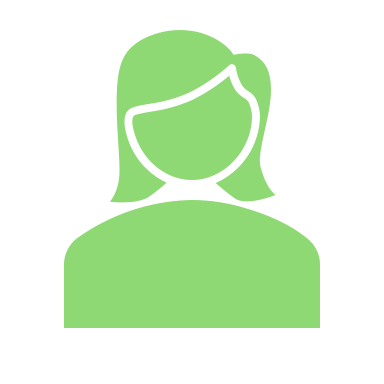

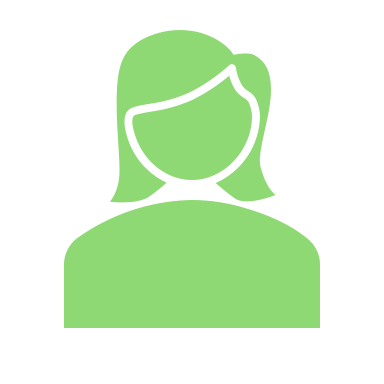

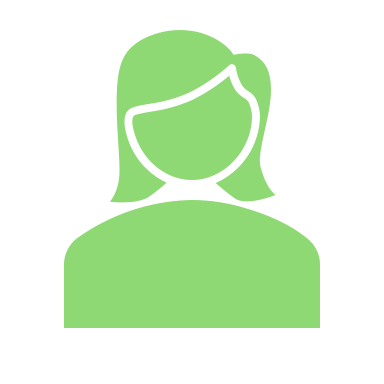

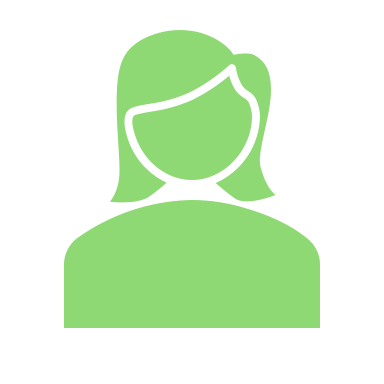

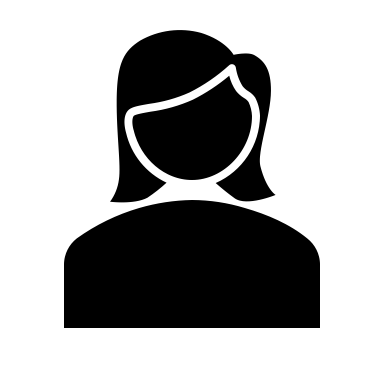

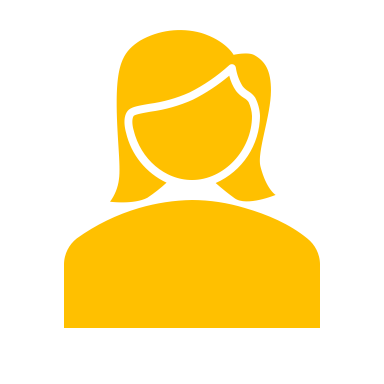

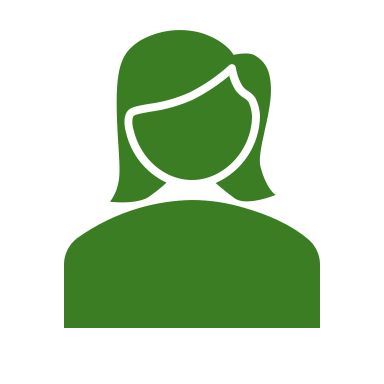


49.7% Junior doctors

21.8% Nurses and midwives

6.1% Respiratory therapist

1.6% Other clinicians (unspecified)

7.4 % Doctors (combination of junior and senior)

12.4% Other clinicians (combination of nurses, nurse practitioners, doctors, and respiratory therapist)

0.9% Paramedics

0.1% Advance practice clinicians


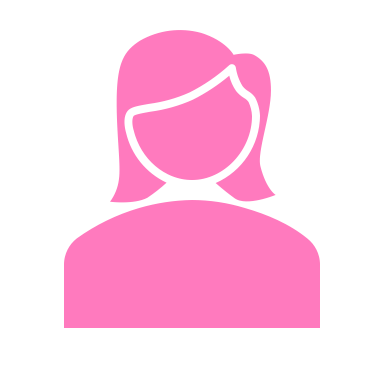

Supplement: Supplementary Data 7 [file mmc7.docx]
